# Optimizing Botulinum Toxin A Administration for Forehead Wrinkles: Introducing the Lines and Dots (LADs) Technique and a Predictive Dosage Model

**DOI:** 10.3390/toxins16020109

**Published:** 2024-02-17

**Authors:** Kamal Alhallak

**Affiliations:** 1 Albany Cosmetic and Laser Centre, Edmonton, AB T6V 1J6, Canada; kalhallak@albanylaser.ca or alhallak@ualberta.ca; Tel.: +1-(587)520-2835; 2 Alberta Cosmetic Pharmacist Association ACPA, Edmonton, AB T6V 1J6, Canada

**Keywords:** botulinum toxin type A, forehead wrinkles, nerve pathways, cosmetic dermatology, personalized treatment

## Abstract

This study introduces the Lines and Dots (LADs) technique, a new approach for administering botulinum toxin type A (BoNT-A) in treating forehead wrinkles. (1) Background: BoNT-A application patterns in the forehead often rely solely on the anatomy of the frontalis muscle. The LADs technique proposes a combination of anatomical features with nerve pathways. (2) Methods: The technique employed a grid system aligned with the supraorbital and supratrochlear nerve pathways and used an electronic acupuncture pen for validation. This study analyzed treatment outcomes for efficacy and safety and proposed a predictive model for BoNT-A dosage. (3) Results: LADs was associated with a high satisfaction rate and low side effect incidence. The predictive model followed BoNT-A Units=0.322×Muscle Pattern Code+1.282×Line Type Code+2.905×Severity Pre-Treatment+3.947. (4) Conclusions: The LADs technique offers an alternative approach to treating forehead wrinkles, optimizing efficacy while minimizing the BoNT-A dose required.

## 1. Introduction

The field of aesthetic medicine is constantly evolving [1], especially in the use of botulinum toxin type A (BoNT-A) for treating facial signs of aging [2,3,4,5]. Forehead lines are a common concern among many adults, and there’s an increasing need for treatment methods that are both effective and consider each patient’s unique facial characteristics [6,7].

The anatomy of the frontalis muscle plays a pivotal role in our understanding of forehead aesthetics [8,9,10]. As the primary muscle involved in forehead expressions, it is responsible for a range of movements, notably the raising of the eyebrows [11,12,13,14]. The activity of the frontalis muscle leads to the formation of horizontal lines across the forehead. These lines are not merely a universal sign of aging but vary significantly in their appearance and depth from individual to individual [15], influenced by factors such as genetic predisposition [16], lifestyle [17], and habitual facial expressions [18].

Given its expansive coverage from the eyebrows to the hairline, the frontalis muscle presents a unique challenge in aesthetic treatments [19]. Uniformly treating this muscle can overlook the importance of its movements and the varying depth of lines it creates. Moreover, such an approach might expose the patient to unnecessary pain and to excessive botulinum toxin A (BoNT-A) due to injections into the aponeurotic tissue rather than into the muscle belly [20]. Therefore, a one-size-fits-all approach falls short in addressing these individual variations [21,22,23,24].

A good understanding of the frontalis muscle’s anatomy and function is crucial for dose prediction and for developing treatment methods that not only smooth out wrinkles but also preserve the natural expressiveness of the forehead [8,25,26,27]. The dose of BoNT-A for forehead wrinkles has traditionally relied on practitioners’ subjective assessment [28,29], but there is an increasing need for objective, data-driven approaches. Predicting accurate BoNT-A doses for forehead lines is crucial for optimal results and safety. Variabilities in anatomy and individual patient goals call for personalized treatment strategies.

In addressing these variations, our approach involves using the natural superficial pathways of the supraorbital (SON) and supratrochlear (SOT) nerves on the middle or upper margin of the frontalis muscle as guides [30,31,32]. While these nerves are primarily sensory for forehead skin [33,34], their anatomical paths may provide valuable landmarks for precisely targeting the frontalis muscle. By aligning our injections with these nerve pathways, we can ensure that BoNT-A is injected close to areas rich in muscle endplate zones located in the middle and upper margins of the frontalis muscle [35].

This method may offer several potential benefits. Firstly, it allows for a reduction in the total amount of BoNT-A used, as the precision of the injections means that smaller quantities can be more effective. Secondly, the risk of unwanted side effects may be minimized, as the injections are less likely to impact areas not intended for treatment. Lastly, it is coupled with a predictive model for dose estimation based on factors like muscle pattern, line type, and pre-treatment wrinkle severity represents a significant advancement. This approach aims to standardize treatments, reduce outcome variability, and align interventions with each patient’s specific needs, marking a shift towards evidence-based, individualized care in aesthetic medicine.

## 2. Results

### 2.1. Validation of the Nerve Path

The validation of the LADs technique for predicting the paths of the supraorbital nerve (SON) and supratrochlear nerve (SOT) using an electronic acupuncture pen yielded excellent results. Upon applying the pen to the points outlined by the LADs grid, there was a consistent production of an audible buzzing sound along with corresponding numerical readings. These readings were indicative of nerve proximity, with higher intensities marking the precise locations of the SON and SOT nerve paths as predicted by the LADs technique.

Conversely, points that fell outside the LADs grid showed few to no pen readings, signaling a lack of proximity to nerve paths. This clear distinction in pen readings between the targeted and non-targeted areas provided substantial evidence of the technique’s accuracy.

### 2.2. Evaluation of Treatment Efficacy and Satisfaction

**Figure 1:**
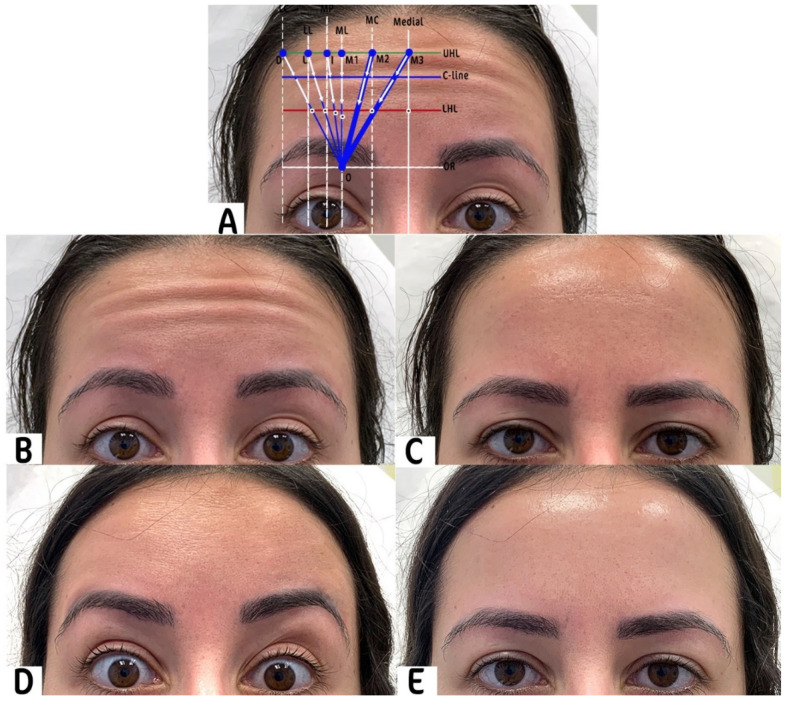
This is a comparative analysis of forehead wrinkles pre and post BoNT-A treatment using the Lines and Dots (LADs) technique. (**A**) LADs grid overlay: pre-treatment frontal view of the patient with the LADs technique grid superimposed on guide treatment areas. (**B**) Maximal forehead contraction pre-treatment: display of forehead at maximum contraction before BoNT-A, showing Grade 2 wrinkles as per the MAS. (**C**) Relaxed forehead pre-treatment: the patient’s forehead at rest before BoNT-A, exhibiting fine lines corresponding to Grade 1 on the MAS. (**D**) Maximal forehead contraction post-treatment: forehead appearance at maximum contraction 2 weeks following BoNT-A, showing no visible wrinkles, achieving Grade 0 on the MAS. (**E**) Relaxed forehead post-treatment: post-BoNT-A relaxed state of the forehead with no visible lines, maintaining a Grade 0 on the MAS, indicative of excellent treatment response.

After administering the Lines and Dots technique (LADs) for BoNT-A treatment, we conducted a comprehensive evaluation of the patients’ pre- and post-treatment images. This assessment utilized the Merz Aesthetics Scale (MAS) to objectively compare the severity of forehead wrinkles before and after the intervention by the principal investigator. The results of this evaluation are exemplified in one patient, as depicted in Figure 1.

The efficacy of the LADs technique for treating forehead wrinkles and patient satisfaction were analyzed across different frontalis muscle patterns. Table 1 is a summary of the findings.

Further detailed outcomes, including patient-specific efficacy, dosage, reported side effects, and satisfaction scores using the LADs technique, are comprehensively presented in Supplementary Table S1.

In this study, a series of correlation analyses were conducted to explore the relationships between the complexity of frontalis muscle patterns and the doses of botulinum toxin A (BoNT-A) administered. Muscle patterns were ordinally coded for complexity: ‘Full’ as 4, ‘V-shaped’ as 3, ‘Central’ as 2, and ‘Lateral’ as 1. Both Pearson and Spearman correlation analyses were utilized to determine the association between these complexity codes and the BoNT-A units used. Furthermore, binary codes were assigned to ‘Static’ (1) and ‘Dynamic’ (0) line types, and a composite severity score was created by weighting these codes according to the severity of the pre-treatment condition. Spearman’s rank correlation was employed to assess the monotonic relationship between this composite severity score and BoNT-A units. This analysis provided insights into the influence of combined factors of line type and severity on treatment dosage, enhancing the understanding of treatment patterns in clinical practice.

The prediction of the number of botulinum toxin A (BoNT-A) units is given by Equation (1):

(1) BoNT-A Units=0.322×Muscle Pattern Code+1.282×Line Type Code+2.905×Severity Pre-Treatment+3.947

where:

- Muscle Pattern Code is coded as Full (4), V-shaped (3), Central (2), or Lateral (1);
- Line Type Code is coded as Static (1) or Dynamic (0).

The model’s performance metrics are:

- Mean Squared Error (MSE): 0.807;
- R-squared (R^2^): 0.935.

## 3. Discussion

The Lines and Dots (LADs) technique introduces a proposed approach to administering BoNT-A for forehead wrinkle treatment. In addition to analyzing the pattern of the frontalis muscle, LADs uses sensory supraorbital and supratrochlear nerve paths to enhance the results of BoNT-A injections and reduce side effects. 

While acknowledging that sensory and motor nerve paths differ, our approach using sensory nerve pathways might also be rich in motor endplates, suggesting that targeting these areas could lead to more efficient BoNT-A use. An electronic acupuncture pen like the LY-508B can help in identifying acupuncture points by measuring of the skin’s electrical properties such as electrical impedance [36]. When the pen is applied to the skin, it emits a low-level electrical current. Acupuncture points have lower electrical resistance compared to surrounding skin, so when the pen encounters an acupuncture point, there is a change in conductivity [37]. This change is typically indicated by the pen through an audible signal, a visual cue, or a change in the sensation felt by the patient, guiding the practitioner to precise points for treatment or diagnosis [38,39,40,41]. The use of the electronic acupuncture pen showed that the points identified by the LADs technique were associated with lower electrical resistance and possibly a higher density of motor endplates.

The LADs technique was designed to adapt to various frontalis muscle patterns. This adaptability is achieved by incorporating additional landmarks, such as the SON and SOT, along with considering factors like the types and severity of wrinkles. This adaptability underscores the technique’s potential in addressing individual patient needs.

It has been shown that targeting areas rich in muscle endplate zones represents a strategic advancement in maximizing the therapeutic potential of BoNT-A [42,43]. By carefully administering reduced doses of BoNT-A directly into these key areas, the method could enhance treatment efficacy while concurrently reducing the likelihood of side effects, such as unintended weakening of surrounding muscles [42,44]. The average number of units of BoNT-A using the LADs technique was 13.2 ± 3.8, which is a significant decrease from what was reported previously (20.7 ± 7.3 units) [45]. The T-test yielded a t-statistic of −9.186 and a highly significant *p*-value of approximately 3.78 × 10^−12^. This extremely low *p*-value (far below the conventional threshold of 0.05) indicates that there is a statistically significant decrease in the average BoNT-A units administered.

Several studies provided insights into the frontalis muscle’s anatomical and physiological characteristics. Measurements indicate a gradual lateral reduction in frontalis muscle thickness from approximately 1.80 mm, identified at the intersection of the pupil’s midline with the horizontal plane connecting the metopion and glabella, to about 1.61 mm at the juncture aligned with the lateral canthus. Further laterally, at the convergence with the line from the lateral orbital rim and the aforementioned horizontal plane, the thickness diminishes to a mere 0.11 mm [46,47,48]. One study identifies that motor endplates, essential for motor function, are predominantly located in the middle and upper portion of the frontalis [35]. Additionally, the absence of superficial fascia in the frontalis muscle suggests that superficial BoNT-A injections could be sufficiently effective, potentially enhancing patient comfort. 

Sihler staining, a technique that selectively stains myelin sheaths, offers a clear visualization of nerve pathways while keeping the nerves intact. This method is extensively recognized in mapping the distribution of motor neurons [49]. Imaging showed that the facial nerve’s temporal branch (TBFN) courses superomedially towards the upper eyelid. Moreover, the Sihler image demonstrated a sunken V shape, and there were no neural distributions. Furthermore, imaging showed a substantial aggregation of deep TBFN terminals that correspond with the location of the SON [20].

**Figure 2:**
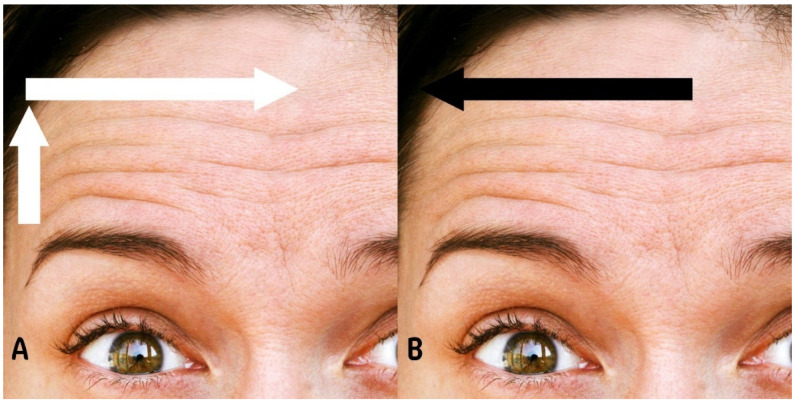
This figure shows (**A**) demonstration of botulinum toxin A (BoNT-A) dose distribution, indicating an increasing pattern from lateral to medial and from lower to upper regions of the forehead. (**B**) Illustration of BoNT-A injection depth progression, showing an increase from medial to lateral across the forehead.

These findings support our proposed techniques that utilize the SON and SOT to optimize efficacy and minimize risks such as brow ptosis. Moreover, they support our suggestions regarding the depth of injection and the dosage of BoNT-A, as shown in Figure 2. 

A possible advantage of this technique is targeting areas with a dense presence of TBFN terminals, which contribute to the formation of forehead wrinkles while selectively avoiding the lateral regions of the frontalis muscle. Therefore, our approach may significantly reduce the likelihood of eyebrow drooping.

In regard to the injection technique, the LADs technique proposes a retrograde linear injection above the UHL, complemented by superficial hypodermic pinpoint injections closer to the UHL. This approach may contribute to the safety of BoNT-A injections as it has been shown that retrograde linear injections are superior in reducing forehead lines [50,51]. However, superficial hypodermic injections are safer, particularly in the lower part of the frontalis muscle [12,52,53].

This study attempts to develop a predictive equation for estimating the required amount of BoNT-A. The proposed model, with an R-squared value of 0.935, explains a significant portion of the variability in BoNT-A units, indicating a strong fit. This model, focusing solely on pre-treatment factors, serves as a valuable tool for practitioners, aiding in dosage estimation based on individual patient characteristics. However, it is crucial to view it as a supplementary guide rather than a replacement for clinical judgment. The actual dosage may still require adjustments based on individual patient responses and practitioner expertise.

Introducing this predictive equation into clinical practice could help streamline the decision-making process for practitioners, potentially reducing the incidence of over- or under-treatment with BoNT-A. However, it is important to conduct further validation through larger-scale studies and clinical trials to confirm its efficacy and applicability across diverse patient populations.

Acknowledging the limitations of this study, particularly the small sample size and the absence of a control group, is essential. These limitations temper the findings’ generalizability. Moreover, our study did not encompass the documentation of patients’ perceptions of pain, a crucial aspect in the context of cosmetic procedures. This omission is a notable limitation, as understanding and minimizing patient discomfort is key to the overall success of any cosmetic treatment. Future research, encompassing larger cohorts and control groups, is necessary to validate the LADs technique’s efficacy and safety further. Long-term follow-up would also be beneficial in assessing the persistence of treatment effects and patient satisfaction over time. Despite these limitations, the LADs technique represents a promising development in cosmetic dermatology, offering a nuanced and personalized method for treating forehead wrinkles with BoNT-A. Its focus on individual anatomical variations and functional needs aligns with the evolving trend towards more customized, patient-centered cosmetic treatments.

## 4. Conclusions

The Lines and Dots (LADs) technique, as explored in this study, signifies a notable advancement in the personalized application of BoNT-A for forehead wrinkles. By innovatively leveraging sensory nerve paths and employing a targeted grid system, the technique demonstrates a potential to enhance the precision and efficacy of BoNT-A treatments. The incorporation of a proposed predictive equation for dosage estimation represents a significant stride towards tailoring treatments to individual anatomical and functional variations, which is key to achieving natural and satisfactory outcomes. While the results are promising, the technique’s full potential and applicability necessitate further validation through expanded research and clinical trials. This study, despite its limitations, contributes to the evolving landscape of cosmetic dermatology, emphasizing the importance of customized approaches in enhancing patient-centric care.

## 5. Materials and Methods

### 5.1. Study Design

This research was conducted as a prospective observational study at our aesthetic medicine center. Over a period of six months, we recruited 30 volunteers who specifically sought to reduce the appearance of horizontal forehead lines. These patients were interested in and treated with the Lines and Dots (LADs) technique, which is the standard method used in our practice for addressing such concerns.

It is important to note that the use of the LADs technique was independent of the participants’ consent to be included in this study. However, their participation in terms of data collection was entirely voluntary and based on informed consent. No incentives were offered for participation in this study. The primary data collected included the amount of BoNT-A used, the severity of forehead lines before and after treatment, and any side effects observed. This approach allowed us to objectively assess the effectiveness of the LADs technique in a real-world clinical setting.

The data did not encompass any personally identifiable information such as ethnicity, age, or other private details. Patients were given the option to consent to or opt out of the use of their treatment photographs for research purposes. This respect for patient autonomy and privacy was a key component of our ethical approach to the study [54].

Our study targeted volunteers aged 30–55 years, specifically interested in addressing horizontal forehead lines using the Lines and Dots (LADs) technique. Inclusion criteria required participants to be in good general health, provide informed consent for the use of their treatment data and, optionally, for treatment photographs. Participants also needed to agree to a structured follow-up schedule, including phone check-ins after 2 days and 7 days, followed by an in-person follow-up after 4 weeks. Exclusion criteria included individuals with conditions contraindicating BoNT-A use (such as neurological disorders), pregnant or lactating women, those with a history of hypersensitivity to BoNT-A, and anyone who had undergone facial cosmetic procedures within the previous 6 months. Additionally, those unwilling or unable to adhere to post-procedure care instructions or the follow-up schedule were not considered for the study.

### 5.2. Assessment of Frontalis Muscle and Horizontal Lines

Before initiating the LADs technique, a comprehensive assessment is conducted for each patient to ensure optimal outcomes in the treatment of forehead wrinkles using BoNT-A. This meticulous assessment process begins with evaluating patient eligibility for BoNT-A treatment, where factors such as the severity and type of forehead wrinkles, muscle hyperactivity, and potential contraindications to BoNT-A use are carefully examined. The patient’s facial anatomy is then thoroughly analyzed, with a particular focus on the frontalis muscle. This involves studying the muscle’s strength, contraction patterns, and the presence of asymmetries or static lines at rest, essential for determining the precise placement of injections as per the LADs methodology.

During the consultation, the patient’s aesthetic goals and expectations are discussed in depth. This step is pivotal to customizing the treatment plan to achieve the desired outcome while ensuring a natural appearance. Photographic documentation of the patient’s face, captured from multiple angles, is employed as a vital tool for assessing the pre-treatment condition and aiding in the communication process with the patient.

**Figure 3:**
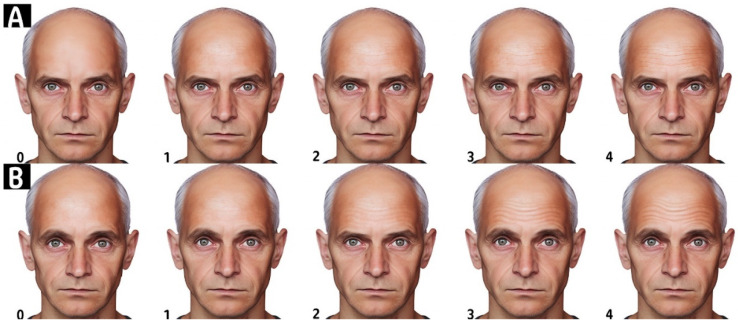
This figure illustrates the Forehead Lines Grading Scale. This figure is divided into two sections: (**A**,**B**). Section (**A**) demonstrates the scoring of the photonumeric scale for resting or static forehead lines, while section (**B**) illustrates the scoring for hyperkinetic or dynamic forehead lines. The scale is a 5-point photonumeric rating system, where each point represents a different severity of wrinkles: 0 indicates no wrinkles, 1 represents no wrinkles present at rest but fine lines with facial expression, 2 is for fine lines present at rest and deep lines with facial expression, 3 signifies fine wrinkles present at rest and deeper lines with facial expression, and 4 denotes deep wrinkles at rest and deeper furrows with facial expression. Artwork by Kamal Alhallak.

The Standard Evaluation of the Patient (The Merz Scale), depicted in Figure 3, provides a standardized method to objectively assess the severity of forehead wrinkles. It is a 5-point photonumeric rating system specifically designed to differentiate between resting (static) and hyperkinetic (dynamic) forehead lines. This scale enables clinicians and researchers to evaluate the presence and depth of forehead lines both when the face is at rest and during facial expressions. A score of 0 indicates the absence of wrinkles, while a score of 1 signifies that no wrinkles are visible at rest, yet fine lines appear with facial movements. A score of 2 denotes fine lines at rest that evolve into pronounced lines upon facial expressions. The presence of fine wrinkles at rest that transition into deeper lines with movement is graded as a 3. The highest score of 4 is reserved for those with deeper wrinkles while the face is at rest, which further deepen into pronounced furrows during facial expressions. This grading scale is pivotal in evaluating the progression of forehead lines and the effectiveness of various dermatological treatments.

This comprehensive evaluation, encompassing both clinical and aesthetic aspects, is integral to the success of the LADs technique, ensuring that each treatment is uniquely adapted to the patient’s individual anatomical and functional characteristics.

### 5.3. The Lines and Dots Technique

#### Locating the Supraorbital and Supratrochlear Nerve

**Figure 4:**
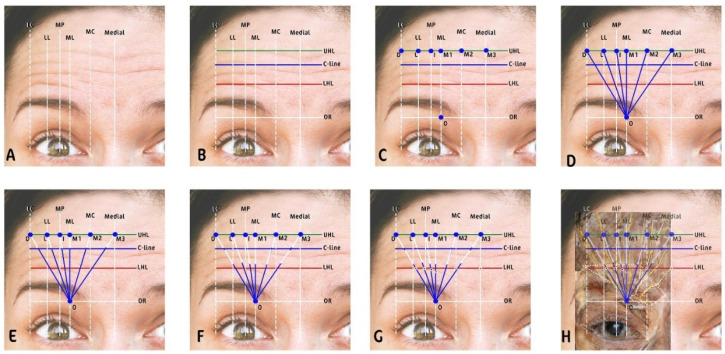
This figure is a visualization of the Lines and Dots Technique (LADs) for botulinum toxin injection. (**A**) Vertical lines are drawn corresponding to key facial landmarks, i.e., lateral canthus (LC), mid-pupil (MP), medial limbus (ML), and medial canthus (MC), establishing a framework for injection planning. (**B**) Horizontal lines are added, i.e., the uppermost horizontal line (UHL), the C-line marking the central forehead crease, and the lowest horizontal line (LHL), providing additional structure to the injection grid. (**C**) The intersection of vertical and horizontal lines creates grid points which serve as potential injection sites for the LADs technique. (**D**) Nerve paths are identified, with blue lines tracing the expected courses of supraorbital nerve (SON) and supratrochlear nerve (SOT) branches. (**E**) The upper injection pattern marked with white arrows, with blue dots indicating where injections are made to target the upper portions of the forehead along the nerve paths. (**F**) The lower injection pattern marked with white arrows, with blue dots marking the injection sites below the C-line to address the lower forehead and eyebrow region. (**G**) Intradermal injection points are indicated, demonstrating the precise locations for the BoNT-A to be administered. (**H**) An overlay of the LADs grid is superimposed over a cadaver dissection image to illustrate the correlation between the theoretical grid and the actual anatomical nerve locations, validating the LADs technique’s anatomical accuracy. Artwork by Kamal Alhallak.

The methodology begins by identifying the following horizontal and vertical lines on the forehead, as shown in Figure 4.

Draw six vertical lines:

- Medial canthus (MC): draw a vertical line upwards from the medial corner of the eye;
- Medial limbus (ML): draw a vertical line upwards from the medial edge of the iris;
- Mid-pupil (MP): draw another vertical line upwards from the midpoint of the pupil when the subject is looking straight ahead;
- Lateral limbus (LL): draw a vertical line upwards from the lateral edge of the iris;
- Lateral canthus (LC): draw a vertical line upwards from the lateral corner of the eye;
- Medial line: this represents mid-sagittal symmetry line between the right and left sides of the face.

Draw four horizontal lines:

- Suborbital ridge (SOR): this horizontal line runs along the bony ridge of the eye socket;
- Lowest horizontal line (LHL): draw the lowest horizontal forehead crease visible when the eyebrows are elevated;
- C-line: draw a horizontal line along the second prominent horizontal forehead wrinkle.
- Uppermost horizontal line (UHL): draw the highest visible line on the forehead when the eyebrows are elevated.

Identify intersection points:

- O: intersection of SOR and ML;
- D: intersection of UHL and LC;
- L: intersection of UHL and LL;
- I: intersection of UHL and MP;
- M1: intersection of UHL and ML;
- M2: intersection of UHL and MC;
- M3: intersection of UHL and the medial line.

Nerve path approximation:

- Deep SON (D-SON) branch path: connect O to D, illustrating the path of the deep branch;
- Lateral SON (L-SON) branch path: draw a line from O to L, representing the SON lateral branch;
- Intermediate SON (I-SON) branch path: connect O to I, showing the path of the intermediate branch;
- Medial SON (M-SON) branch path: connect O to M1, indicating the SON medial branch;
- Lateral SOT (L-SOT) branch path: connect O to M2, indicating the SOT medial branch;
- Medial SOT (M-SOT) branch path: connect O to M3, indicating the SOT lateral branch.

Ensuring the accuracy of nerve pathway identification is critical for the success of the LADs technique. To validate the paths of the supraorbital nerve (SON) and supratrochlear nerve (SOT), we employed an innovative approach by using an electronic acupuncture pen (Electronic LY-508B Acupuncture Pen, ACUPRESSURE HEALTH CARE SYSTEM, China) as a surrogate for a conventional EMG device. This portable and accessible instrument was utilized to locate and validate the nerve paths. The procedure involved placing a grounding electrode in the patient’s hand, while the tip of the acupuncture pen was applied to the marked points on the forehead. As the pen approached the vicinity of a nerve, it produced an audible buzzing sound and a numerical reading indicative of nerve proximity. The intensity of the response correlated with the closeness to the nerve path, providing a practical and immediate means of verification. Due to the short distance between the points, the sensitivity was set at level 2 out of 10 to improve accuracy and decrease false positivity, as shown in Video S1.

### 5.4. BoNT-A Reconstitution

A total of 100 units of freeze-dried OnabotulinumtoxinA (Botox Cosmetic, Allergan INC., Irvine, CA, USA) was reconstituted with 4 mL of sterile solution of 0.9% sodium chloride for injection following common aseptic techniques. This dilution yields 5 units/0.2 mL. The high dilution allows practitioners to distribute low doses of BoNT-A in a large area.

### 5.5. Injection Technique

The injection followed the paths illustrated by the white vectors in the accompanying figure, using a 1 mL Luer-lock, low-dead-space syringe (HENKE-JECT, HENKE SASS WOLF, Tuttlingen, Germany) and low-dead-space 0.2 mm 34 gauge × 9 mm needles (The Invisible Needle TM, TSK Laboratory, Tochigi-Kan, Japan). The procedure began from the lateral aspect of the forehead and progressed medially. The initial injection, starting at point D, followed the path of the deep branch of the supraorbital nerve (SON). Utilizing the full length of the needle, the practitioner advanced the BoNT-A along this nerve path until reaching the C-line, angling the needle at approximately 15 degrees to facilitate intradermal delivery.

Subsequent injections followed a similar protocol, beginning from the endpoint of the preceding injection at the C-line and continuing along the same nerve path, terminating at the lowest horizontal line (LHL). The remaining injections, starting respectively from points L, I, M1, M2, were administered with careful consideration of the direction and depth to remain consistent with the nerve path trajectory. For areas below the LHL, the LADs technique incorporates a microdose pinpoint approach as shown in Video S2. The M3 point should be disregarded unless the patient exhibits a full frontalis pattern.

### 5.6. Customized BoNT-A Dose

The general rule for the LADs technique is that the dose increases lateral to medial and decreases upper to lower, while injection depth increases medial to lateral, as shown in Figure 3.

Therefore, injections from point D will have the lowest dose and deepest injection, while injections at point M3 will have the highest dose, but the most superficial injection. Lines originating from equivalent points on the right and left and side should receive equal doses, unless the practitioner aims to fix some asymmetry. The total dose of BoNT-A should be customized for each patient depending on several factors such as the size of the forehead, the severity of the lines, and the patient’s expectations.

### 5.7. Statistical Analysis

Statistical analyses were performed utilizing Statistical Package for the Social Sciences software version 24 (SPSS^®^, International Business Machines Corp., Armonk, NY, USA). A *p*-value threshold of <0.05 was adopted as the criterion for statistical significance in reporting results.

A series of correlation analyses were conducted to investigate the relationships between various characteristics of frontalis muscle patterns and the administered doses of botulinum toxin A (BoNT-A). Initially, we coded frontalis muscle patterns ordinally based on their complexity (‘Full’ as 4, ‘V-shaped’ as 3, ‘Central’ as 2, ‘Lateral’ as 1) and performed both Pearson and Spearman correlation analyses to determine the strength and direction of their associations with BoNT-A units. To explore the influence of line type, we assigned binary codes to ‘Static’ (1) and ‘Dynamic’ (0) line types. We further weighted these codes by the severity of the pre-treatment condition, creating a composite severity score.

A linear regression model was used to predict the dosage of botulinum toxin A (BoNT-A) based on the characteristics of frontalis muscle patterns. The dataset included variables such as frontalis muscle pattern (coded ordinally as Full = 4, V-shaped = 3, Central = 2, Lateral = 1), line type (coded as Static = 1, Dynamic = 0), and pre-treatment severity (graded on a scale). We excluded the post-treatment severity variable to focus solely on pre-treatment factors.

The data were split into training and testing sets, with 80% of the data used for training and the remaining 20% for testing. A linear regression model from the scikit-learn library in Python was employed for our analysis (Python 3.8.10, Python Software Foundation, Wilmington, DE, USA). The model’s performance was evaluated using the Mean Squared Error (MSE) and the R-squared (R^2^) metrics, providing insights into the model’s predictive accuracy and the proportion of variance in the BoNT-A dosage explained by the model.

## 6. Patents

The author has applied for a trademark registration of the ‘Lines and Dots’ (LADs) technique, underlining its uniqueness and proprietary status in the field of cosmetic dermatology.

## Figures and Tables

**Table 1:** This is a comparative analysis of botulinum toxin type A (BoNT-A) administration across different frontalis muscle patterns: efficacy, dosage, and patient satisfaction.

Frontalis Muscle Pattern	Number of Cases	Average # of Units	Static	Dynamic	Number of Side Effects	Average Satisfaction
**Full**	14	14.92	17.11	10	2	9.79
**V-shaped**	10	12.5	15	10	1	9.9
**Lateral**	3	7	7	7	0	10
**Central**	3	14.33	15	13	0	10

## Data Availability

The datasets presented in this article are not readily available due to privacy concerns and as a condition for exemption from certain requirements. These constraints were established to protect the confidentiality and integrity of the data subjects involved in the study. Requests to access the datasets should be directed to kalhallak@albanylaser.ca.

## Supplementary Materials

The following supporting information can be downloaded at: https://www.mdpi.com/article/10.3390/toxins16020109/s1, Table S1: Comparative Data of BoNT-A Efficacy, Dosage, and Patient Satisfaction Across Frontalis Muscle Pattern; Video S1: This video demonstrates the innovative use of the Electronic LY-508B Acupuncture Pen as a method for validating the nerve pathways critical to the Lines and Dots (LADs) technique. The procedure showcases the placement of a grounding electrode in the patient’s hand and the application of the acupuncture pen on specific marked points on the forehead. Notably, as the pen approaches a nerve, it emits an audible buzzing sound accompanied by a numerical reading, indicative of nerve proximity. The video highlights the pen’s response intensity, correlating to nerve closeness, with the sensitivity set at level 2 out of 10 for enhanced accuracy and reduced false positives; Video S2: This video illustrates the precise application of BoNT-A following the Lines and Dots (LADs) technique. The process begins from the lateral aspect of the forehead, using a 1 mL Luer-lock, low-dead-space syringe and ultra-fine needles for accurate delivery. The initial injection starts at point D, targeting the deep branch of the supraorbital nerve (SON), with the needle angled at approximately 15 degrees for optimal intradermal injection. The video showcases the methodical progression of injections from the endpoint of one nerve path at the C-line to the next, adhering to the illustrated paths for precise targeting. Special attention is given to the direction and depth of injections to ensure alignment with nerve pathways, culminating in the microdose pinpoint approach for areas below the lowest horizontal line (LHL), demonstrating the meticulous nature of the LADs technique.
