# Supplementary material for: Optimizing Botulinum Toxin A Administration for Forehead Wrinkles: Introducing the Lines and Dots (LADs) Technique and a Predictive Dosage Model
Source: Toxins (Basel). 2024 Feb 17;16(2):109. doi: 10.3390/toxins16020109 (PMC10893323; doi:10.3390/toxins16020109)
Supplement: Supplementary file 1 [file toxins-16-00109-s001.zip › toxins-2848602-su.pdf]

Supplementary Materials: Optimizing Botulinum Toxin A Administration for Forehead Wrinkles: Introducing the Lines and Dots (LADs) Technique and a Predictive Dosage Model

Table S1: Comparative Data of BoNT-A Efficacy, Dosage, and Patient Satisfaction Across Frontalis Muscle Pattern

Picipant ID	Frontalis Muscle Pattern	Line Type	Severity Pre-Treatment	Severity Post-Treatment	BoNT-A Units	Reported Side Effects	Patient Satisfaction (0-10)
1	Full	Static	Grade 4	Grade 1	20	None	10
2	Full	Static	Grade 3	Grade 0	15	Mild Swelling	9
3	Full	Static	Grade 4	Grade 1	20	Minor Bruising	8
4	Full	Static	Grade 3	Grade 0	15	None	10
5	Full	Dynamic	Grade 2	Grade 0	10	None	10
6	Full	Static	Grade 3	Grade 1	15	None	10
7	Full	Dynamic	Grade 2	Grade 0	10	None	10
8	Full	Static	Grade 4	Grade 1	18	None	10
9	Full	Dynamic	Grade 2	Grade 0	10	None	10
10	Full	Static	Grade 4	Grade 1	18	None	10
11	V-shaped	Static	Grade 3	Grade 0	15	None	10
12	V-shaped	Dynamic	Grade 2	Grade 0	11	None	10
13	V-shaped	Static	Grade 3	Grade 1	15	None	10
14	V-shaped	Dynamic	Grade 2	Grade 0	11	None	10
15	V-shaped	Static	Grade 3	Grade 0	15	None	10
16	V-shaped	Dynamic	Grade 1	Grade 0	9	None	10
17	V-shaped	Static	Grade 3	Grade 1	15	None	10
18	V-shaped	Dynamic	Grade 1	Grade 0	9	Mild Swelling	9
19	V-shaped	Static	Grade 3	Grade 0	15	None	10
20	V-shaped	Dynamic	Grade 1	Grade 0	10	None	10
21	Lateral	Static	Grade 1	Grade 0	7	None	10
22	Lateral	Dynamic	Grade 1	Grade 0	7	None	10
23	Lateral	Dynamic	Grade 1	Grade 0	7	None	10
24	Central	Static	Grade 3	Grade 1	15	None	10
25	Central	Static	Grade 3	Grade 1	15	None	10
26	Central	dynamic	Grade 3	Grade 1	13	None	10
27	Full	Dynamic	Grade 2	Grade 0	10	None	10
28	Full	Static	Grade 3	Grade 0	15	None	10
29	Full	Static	Grade 4	Grade 1	18	None	10
30	Full	Dynamic	Grade 2	Grade 0	10	None	10
